# Synthesis of Novel Asymmetric Zinc (II) Phthalocyanines Bearing Octadecyloxyl and Glucosyl Groups

**DOI:** 10.3390/molecules14093688

**Published:** 2009-09-18

**Authors:** Pei Zhang, Shufen Zhang, Gang Han

**Affiliations:** State Key Laboratory of Fine Chemicals, Dalian University of Technology, Dalian 116012, China E-Mails: spaceswimer@hotmail.com (P.Z.); hangangxiaoxi@hotmail.com (G.H.)

**Keywords:** carbohydrates, phthalocyanine, asymmetric, deprotection

## Abstract

A novel asymmetric zinc (II) phthalocyanine substituted by one lipophilic octadecyloxyl group and three hydrophilic glucosyl groups was synthesized. Using Q-TOF MS, the major byproducts formed during the deprotection processes were identified. An improved procedure was worked out to convert these byproducts to the desired product in quantitative yield.

## 1. Introduction

Photodynamic therapy (PDT) has attracted increasing attention as a treatment for cancer in recent years [1,2,3,4,5,6,7,8], and photosensitizers play a critical role in this therapy. With a strong absorption at the red light region, the ability to generate singlet oxygen efficiently, and a long excitation wavelength which enables it to act deep under the skin, phthalocyanine is an ideal parent structure for a photosensitizer [9,10]. However, the poor aqueous solubility of phthalocyanine significantly limits its application in PDT. To improve its solubility, an ionic modification of phthalocyanine was made [11], but this failed to give the desired results. The resulting derivative also failed to meet the requirement of selective absorption by tumor cells. Compared to normal cells, tumor cells require more glucose as an energy supply. Additional glucose as a handle might assist penetration into target cells with the help of the functional glucose transporters on the cell membrane [12]. Thus, glucoconjugation of photosensitizers was expected to increase their selective absorption by tumor cells and their transportation through cell membranes. Glucoconjugated phthalocyanine was first reported in 1989 [13], followed in recent years by silicon(IV) phthalocyanines with one or two axial acetal-protected galactose substituents [14], glucose substituted zinc(II) phthalocyanine linked via the anomeric carbon [15], asymmetrical zinc(II) phthalocyanine with four galactose substituents [16], glycosylated zinc(II) phthalocyanines through O or S [17], octasubstituted galactose zinc(II) phthalocyanine [18], and amphiphilic Ni phthalocyanines bearing a hydrophilic galactose head facing six hydrophobic thiohexyl chains [19]. Amphiphilic capacity is an important factor in the design of photosensitizer due to the hydrophobic nature of the lipid membrane [20,21]. In our work, an asymmetrical zinc (II) phthalocyanine bearing three glucosyl groups and an octadecyloxyl group was designed and synthesized. Our synthetic methodology allows ready modulation of the amphiphilic capacity of phthalocyanines by changing the alkoxyl groups, which will provide a base for further studies in the impact of amphiphilic capacity on photosensitizer effects in PDT.

## 2. Results and Discussion

**Scheme 1:**
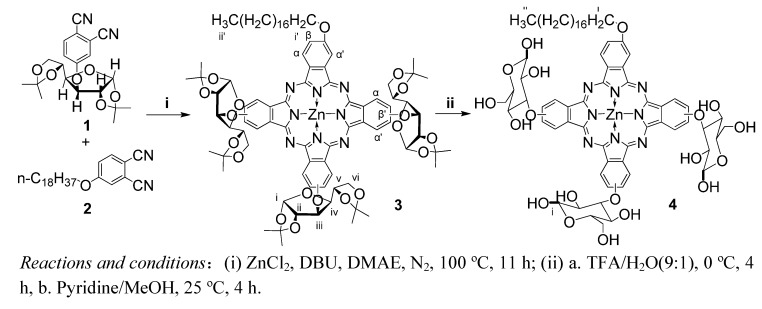
Synthesis of [2(3), 9(10), 16(17)-tris(glucosyl)-23(24)-octadecyloxyl phthalocyaninato] zinc (II) (**4**).

The preparation of asymmetric phthalocyanine **4** is shown in Scheme 1. Firstly, protected glucosyl phthalonitrile **1** (hydrophilic monomer) and octadecyloxyl phthalonitrile **2** (lipophilic monomer) were synthesized by nucleophilic substitution [13]. Phthalocyanine **3** was then obtained by statistical cross-condensation in the presence of zinc chloride and DBU (1,8-diazabicyclo[5.4.0]undec-7-ene) under nitrogen at 100 ºC [22,23]. According to a probabilistic calculation, the highest theoretical yield is reached when the molar ratio of the hydrophilic monomer to the lipophilic monomer is 3:1. The reaction gave a series of phthalocyanines with different numbers of glucosyl and octadecyloxyl groups. The desired product **3** was isolated by silica gel column chromatography eluted with a gradient of ethyl acetate and toluene.

**Figure 1:**
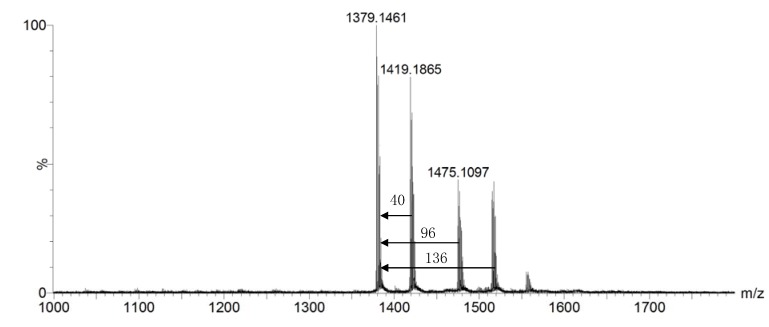
Typical Q-TOF mass spectrum of raw products of compound 3 after deprotection reaction.

The deprotection of compound **3** was initially carried out as follows: a sample of **3** in trifluoroacetic acid (TFA)/H_2_O (9/1 v/vwas stirred in the dark at 20 ºC for 30 min; then the reaction mixture was poured into toluene and rapidly evaporated under reduced pressure [16]. The product mixture was complicated and difficult to purify [23]. Thus it was analyzed by Q-TOF MS directly without further refinement. According to the Q-TOF results shown in Figure 1, in addition to the signal of the desired product **4** (m/z 1,379), there are several signals (m/z 1,419, 1,475 and 1,515) that may be attributed to compound **4** with one isopropylidene residue (M+40), TFA-esterified compound **4** (M+96) and TFA-esterified compound **4** with one isopropylidene residue (M+40+96), respectively. This indicates that the hydroxyl group had been esterified by TFA under the deprotection conditions. After several attempts at optimizing the deprotection conditions including changes in sample concentrations, the ratio of TFA/H_2_O and reaction temperature and time, esterification by TFA still occurred before the deprotection was complete.

TFA esters undergo hydrolysis under alkaline conditions [24]. In our further attempts to optimize the synthesis, this was used to effect the deprotection of compound **3** by a two-step method. First, the sample was treated with TFA/H_2_O (9/1, v/v) to remove the isopropylidene group, then the mixture was stirred in pyridine/MeOH (1/30, v/v) to remove the trifluoroacetyl group. The desired compound **4** was then obtained in quantitative yield by evaporating the solution under reduced pressure.

The definitive characterization of compound **3** and **4** was achieved using Q-TOF MS. As a mixture of isomers, the ^1^H-NMR spectrum of compound **3** in a mixture of CDCl_3_ and pyridine-d_5_ (20/1, v/v) was in agreement with that of the phthalocyanine parent structure bearing protected glucosyl [24] or alkoxyl [25] groups with long carbon chains. It shows three broad signals between 9.45 and 8.65 ppm due to the resonance of Pc α-H or α’-H (Scheme 1) because there are two kinds of substituent linked to phthalocyanine. For the same reason, two broad signals between 7.95 and 7.45 ppm were assigned to the resonances of Pc β-H (Scheme 1). The ^1^H-NMR spectrum of compound **4** in DMSO-d_6_ was consistent with a phthalocyanine structure substituted by deprotected glucosyl [15,16] or alkoxyl [25] groups with long carbon chains, the signals corresponding to proton H-i of the glucosyl moieties (in α- and β- configurations) appeared at 6.75 and 6.95 ppm. UV-Vis spectrum of compound **4** in MeOH was a typical spectrum of phthalocyanine which had a sharp Q-band at 680 nm.

## 3. Experimental

### 3.1. General methods

For column chromatography, E. Merck 60G Silica Gel was used. ^1^H- and ^13^C-NMR spectra were recorded at ambient temperature using a Varian INOVA spectrometer. Chemical shift values were reported in d (ppm) relative to Me_4_Si. ESI-Q-Tof mass spectra (ESIMS) were measured using a Micromass UPLC/Q-Tof Mass Spectrometer. All reagents were of commercial quality and were purified according to general procedures.

### 3.2. Synthesis of [2(3),9(10),16(17)-tris(1,2:5,6-di-O-isopropylidene-a-D-glucofuranosyl)-23(24)-octadecyloxyl phthalocyaninato] zinc (II) *(**3**)*

Phthalonitrile **1** (2.4 g, 6 mmol), phthalonitrile **2** (0.8 g, 2 mmol) and ZnCl_2_ (0.3 g, 2 mmol) in DMAE (25 mL) were stirred at 100 ºC under a N_2_ atmosphere for 12 h. After cooling, the mixture was poured into water (500 mL) and the solid was filtered. The crude product was purified by chromatography over silica gel [eluent: EtOAc/toluene, 80:20] to give **3**. Yield 0.8 g (25%). ^1^H-NMR (CDCl_3_ + pyridine-d_5_): *δ* = 9.45-8.65 and 7.95-7.45 (12H, Pc-H), 6.20 (3H, H-i), 5.46-5.35 (3H, H-iii), 5.06 (3H, H-ii), ***4***.81 (3H, H-v), 4.68-4.48 (3H, H-iv), 4.41-4.27 (8H, H-vi H-i’), 1.80-1.12 (68H, OCH_3_, CH_2_), 0.87 (3H, H-ii’); ^13^C-NMR (CDCl_3_ + pyridine-d_5_): *δ* = 158.85, 140.59, 140.45, 132.89, 131.39, 130.86, 130.21, 129.99, 129.71, 128.83, 128.11, 127.93, 118.55, 112.45, 109.38, 105.65, 82.80, 81.03, 72.60, 69.01, 67.38, 65.51, 62.12, 34.03, 31.91, 30.60, 29.73, 29.33, 29.12, 27.21, 27.03, 26.51, 26.38, 25.66, 25.42, 24.88, 22.66, 19.18, 14.07, 13.68; UV-vis (CHCl_3_) λ_max_ (log ε): 347 (4.89), 616 (4.51), 681 (5.09) nm; HRMS (Q-TOF): m/z = 1620.9790 (M+H)^+^.

### 3.3. Synthesis of [2(3), 9(10), 16(17)-tris(glucosyl)-23(24)-octadecyloxyl phthalocyaninato] zinc (II) *(**4**)*

Phthalocyanine **3** (10 mg, 6.2 mmol) in TFA/H_2_O (9/1, v/v, 4 mL) was stirred in the dark at 0 ºC for 4 h, the reaction mixture was rapidly co-evaporated with toluene (50 mL) under reduced pressure, then the solid was stirred in the mixture DMF (0.1 mL) and pyridine/MeOH (1/30, v/v, 1mL) in the dark at 25ºC for 4 h. The product was obtained by evaporation under reduced pressure. ^1^H-NMR (DMSO-d_6_): *δ* = 9.40-8.80 and 7.94-7.70 (12H, Pc-H), 6.75 and 6.95 (3H, H-i), 5.72-3.45 (32H, Glu-H H-i’) 2.08-1.08 (32H, CH_2_), 0.81(3H, H-ii’); ^13^C-NMR (CDCl_3_ + pyridine-d_5_): *δ* = 158.24, 157.93, 157.62, 157.30, 153.17, 152.50, 140.33, 139.93, 131.28, 128.96, 128.13, 127.75, 122.70, 121.52, 119.82, 118.54, 115.56, 112.58, 109.02, 96.82, 92.55, 83.51, 76.51, 74.42, 72.17, 71.77, 69.65, 68.38, 60.97, 31.11, 28.85, 28.51, 25.58, 21.91, 21.31, 13.76; UV-Vis (MeOH) λ_max_ (log ε): 349 (4.63), 613 (4.25), 680 (4.83) nm; HRMS (Q-TOF): m/z = 1379.3811 (M+H)^+^.

## 4. Conclusions

In summary, an asymmetric zinc (II) phthalocyanine with both lipophilic and hydrophilic groups has been synthesized and characterized. The TFA-esterified byproducts formed during the deprotection were characterized and converted into the final product by a two-step method which might find general application in the removal of isopropylidene groups from similar compounds. On the basis of this synthetic methodology, a series of asymmetric phthalocyanines with different lipophilic groups could be readily synthesized, which should provide great opportunities to study the impact of amphiphilic capacity on the effect of photosensitizers in PDT.
